# Supplementary figures and images for: Denisovan introgression has shaped the immune system of present-day Papuans
Source: PLoS Genet. 2022 Dec 8;18(12):e1010470. doi: 10.1371/journal.pgen.1010470 (PMC9731433; doi:10.1371/journal.pgen.1010470)

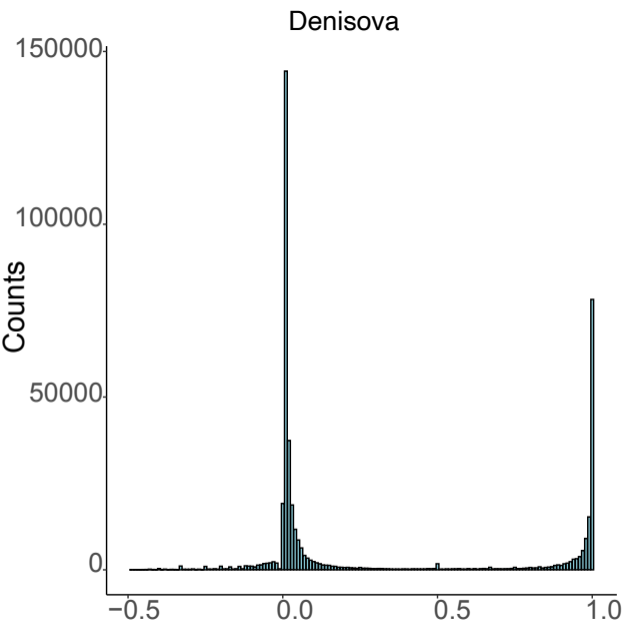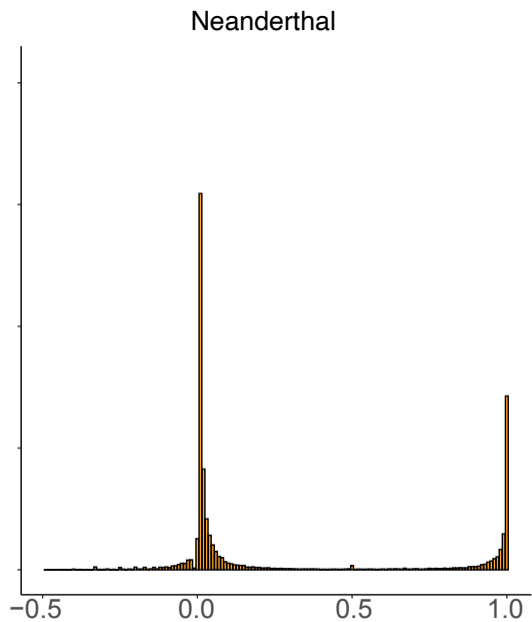

Archaic allele frequency difference between archaic and non-archaic haplotypes

Supplement: S1 Fig — Histogram showing the distribution of the Denisovan and Neanderthal allele frequency differences between archaic and non-archaic haplotypes. Negative values indicate putative archaic variants segregating at higher frequencies within non-archaic haplotypes. (PDF) [file pgen.1010470.s010.pdf]

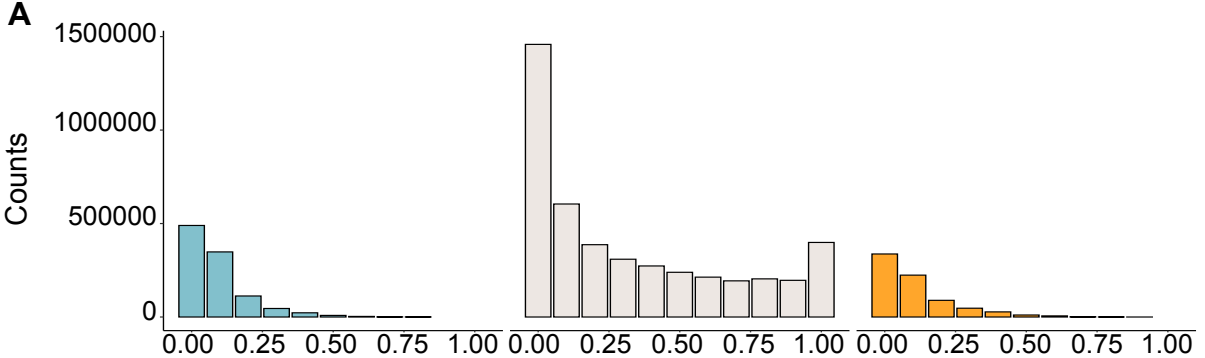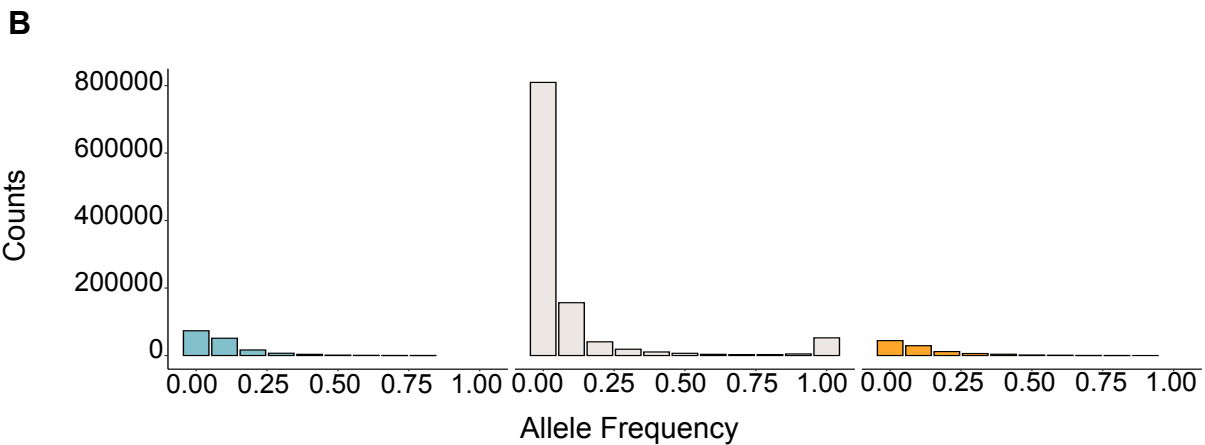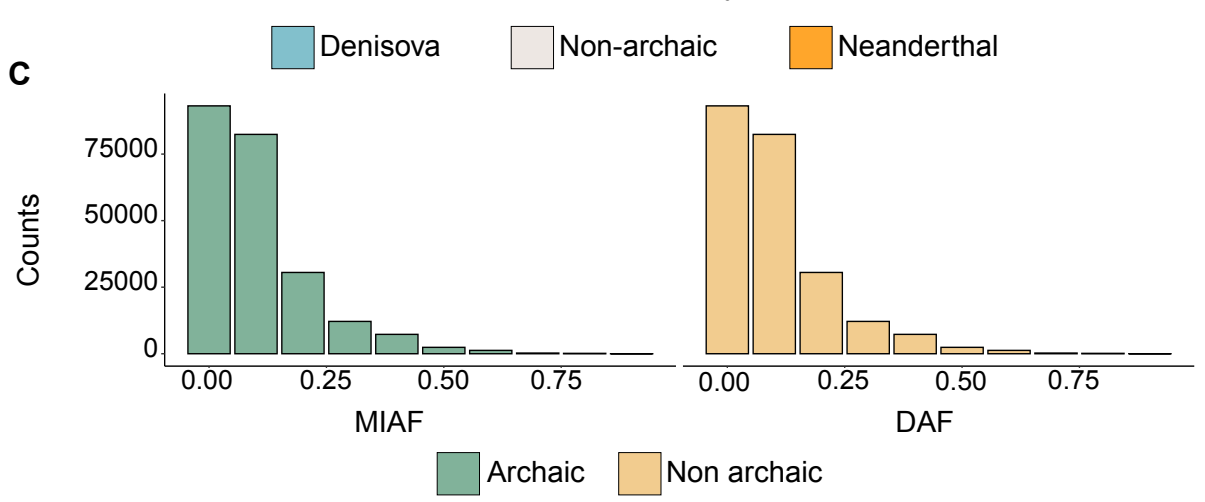

Supplement: S2 Fig — Histogram showing the SFS for Denisovan, Neanderthal and non-archaic variants A) before and B) after the variant filtering steps. C) Histogram showing the SFS for aSNPs (Neanderthal + Denisovan) and the matched background set of naSNPs. (PDF) [file pgen.1010470.s011.pdf]

**A**

B-statistic

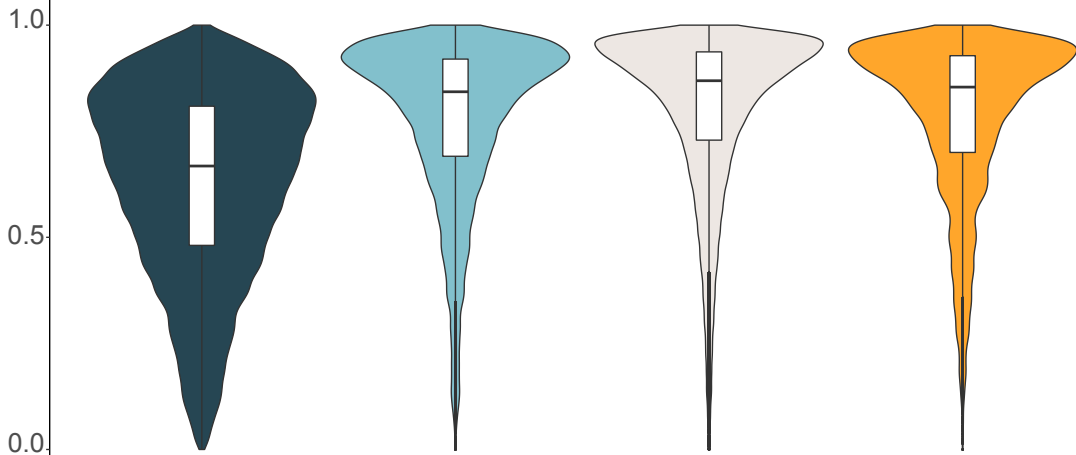**B**

B-statistic

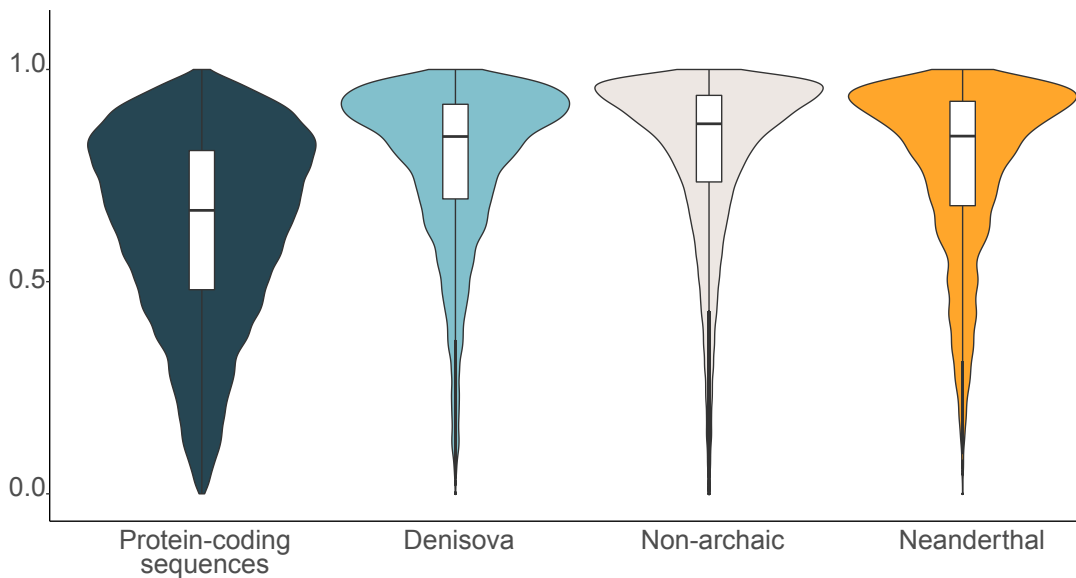

Supplement: S3 Fig — Plots showing the distribution of the B-statistic values for the genomic regions containing the refined set of A) all Denisovan, Neanderthal and non-archaic variants or B) only the common-to-high-frequency SNPs. Lower values indicate higher evolutionary constrains. For visual comparison, the distribution of the B-statistic values for all human protein coding sequences is also shown. Reported p-values are calculated from Mann-Whitney U test. (PDF) [file pgen.1010470.s012.pdf]

**A**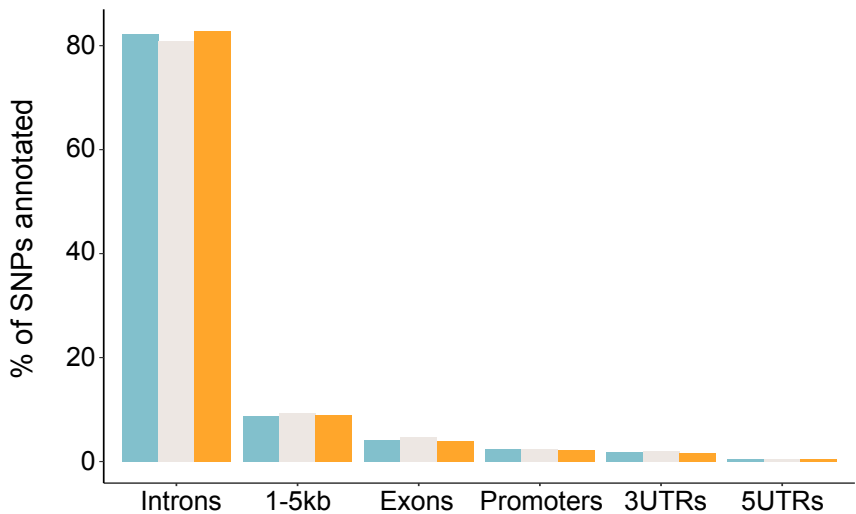**B**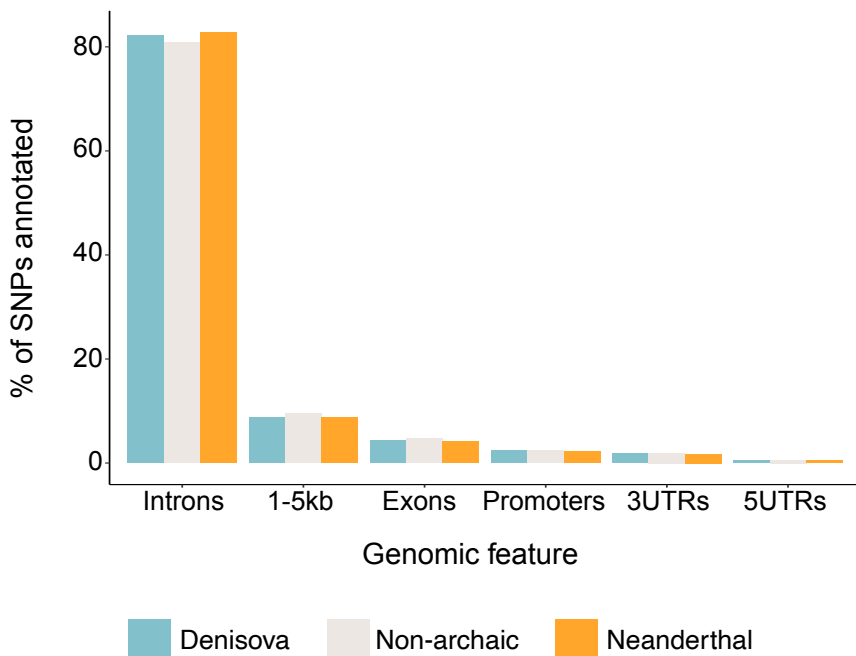

Supplement: S4 Fig — Histogram showing the proportion of A) all aSNPs and naSNPs and B) only common-to-high-frequency variants annotated across multiple genomic elements as reported by annotatr [64]. Percentages are relative to the SNPs annotated across the categories shown. Total numbers of variant annotated: A) 72,761 Denisovan, 44,135 Neanderthal and 114,518 non-archaic; B) 42,334 Denisovan, 27,400 Neanderthal and 68,310 non-archaic. (PDF) [file pgen.1010470.s013.pdf]

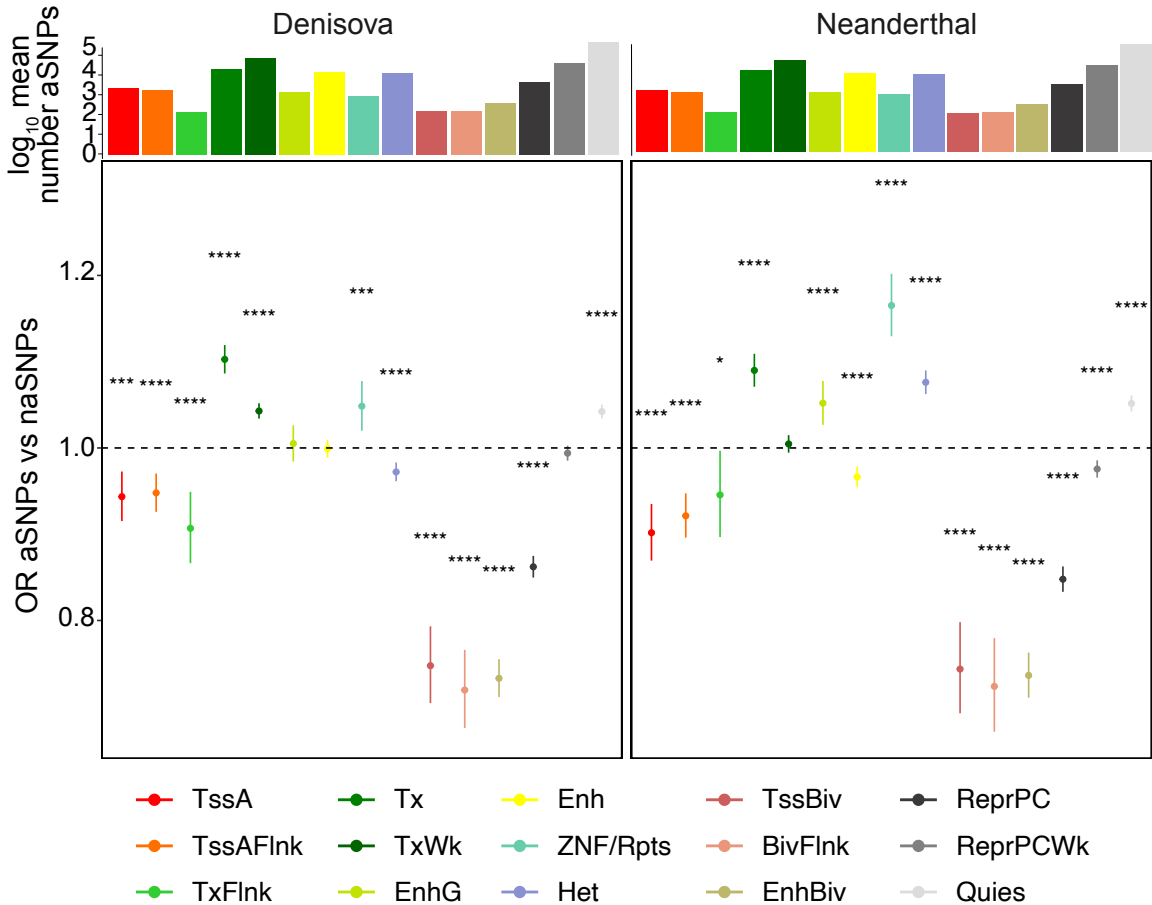

Supplement: S5 Fig — Figure showing the patterns of enrichment across the 15 chromatin states for the entire set of Denisovan and Neanderthal aSNPs relatively to the matched background set of naSNPs. Histograms on top indicate the mean number of variants within each chromatin state calculated across all 111 cell types. Asterisks indicate BH-corrected Fisher’s exact test p-values < 0.05, i.e., **** = p ≤ 0.0001; *** = 0.0001 < p ≤ 0.001; ** = 0.001 < p ≤ 0.01; ** = 0.01 < p ≤ 0.05 (for full statistical results see S6 Table). (PDF) [file pgen.1010470.s014.pdf]

**A**

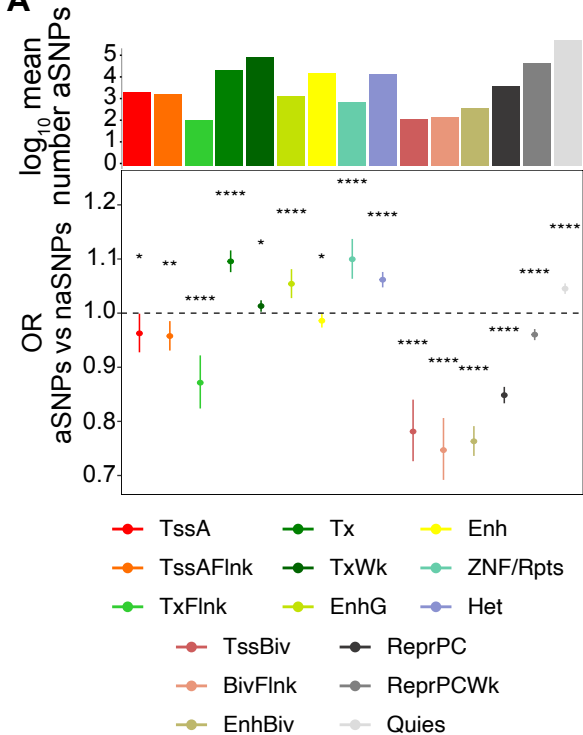

**B**

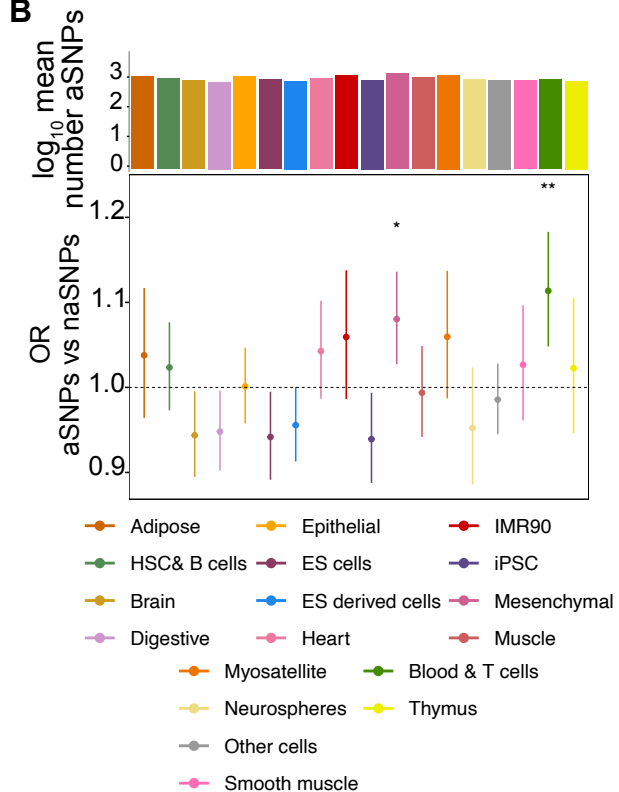

Supplement: S6 Fig — Figure showing the patterns of enrichment across A) the 15 chromatin states for the entire set of Neanderthal aSNPs B) the 18 different tissues for the set of cis-regulatory Neanderthal variants segregating at common-to-high-frequencies in EUR. OR are computed relatively to the matched background set of naSNPs. Histograms on top indicate the mean number of variants annotated within each chromatin state (A) or within each tissue (B). The mean is respectively calculated across all 111 cell types and across the cell types belonging to each tissue. Asterisks indicate BH-corrected Fisher’s exact test p-values < 0.05, i.e., **** = p ≤ 0.0001; *** = 0.0001 < p ≤ 0.001; ** = 0.001 < p ≤ 0.01; ** = 0.01 < p ≤ 0.05. (PDF) [file pgen.1010470.s015.pdf]

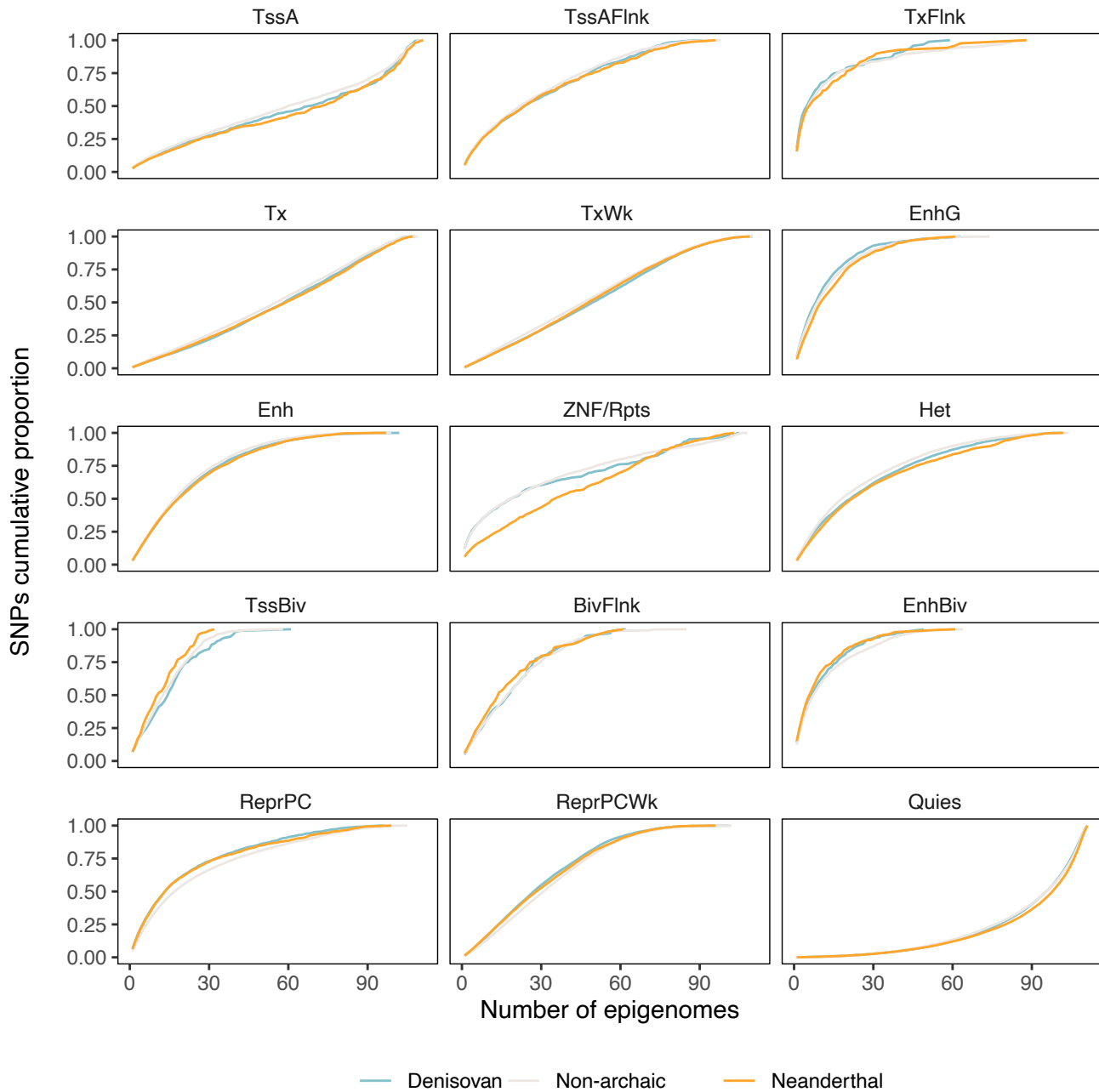

Supplement: S7 Fig — Cumulative proportion of the pleiotropic activity across 111 cell types of each chromatin state-associated element carrying SNPs. (PDF) [file pgen.1010470.s016.pdf]

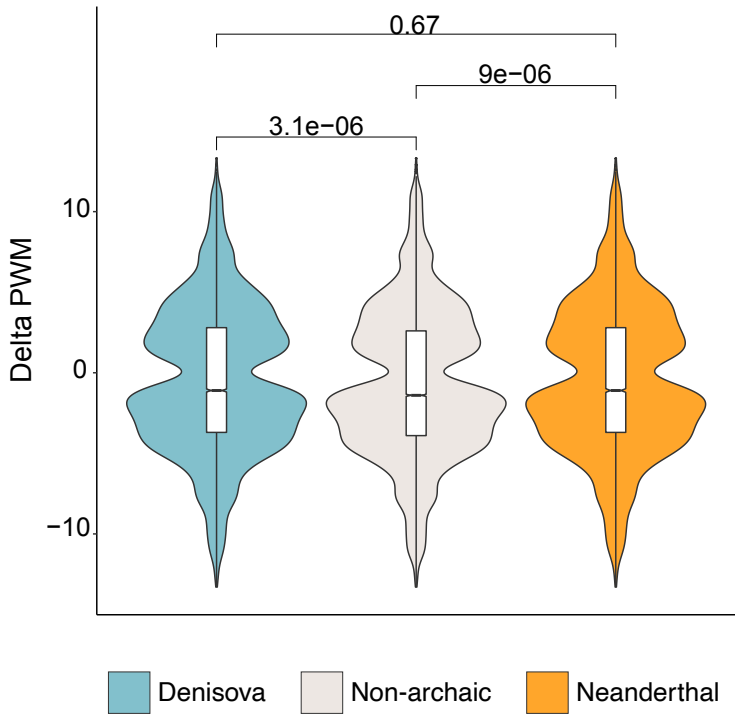

Supplement: S8 Fig — Distribution of the Δ PWM scores for the set of aSNPs and naSNPs. P-values are returned from Wilcoxon test. (PDF) [file pgen.1010470.s017.pdf]

Findley et al 2021

Denisovan

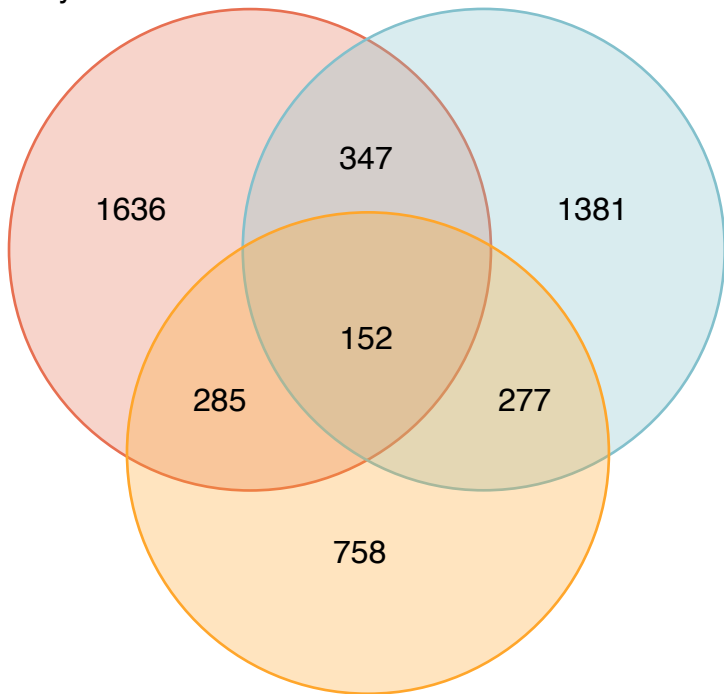

Neanderthal

Supplement: S9 Fig — (PDF) [file pgen.1010470.s018.pdf]
