# Supplementary material for: Denisovan introgression has shaped the immune system of present-day Papuans
Source: PLoS Genet. 2022 Dec 8;18(12):e1010470. doi: 10.1371/journal.pgen.1010470 (PMC9731433; doi:10.1371/journal.pgen.1010470)
Supplement: Attachment — Submitted filename: 20220720_response_to_reviewers.docx [file pgen.1010470.s019.docx]

*We thank all three reviewers for their comments and we agree that our manuscript lacked some details about the methods and controls for potential confounding factors. We have thus incorporated their suggestions and simplified some of the results sections to make reading easier and our conclusions more readily apparent. Below, the reviewer's comments are in plain black text and our responses in* ***bold text.*** *Quoted text from the manuscript is in* ***bold blue.***

**Overview of the overall changes reported in the current version:**

*As pointed by Reviewer #1, the past submission reported the analyses based only on variants segregating at common-to-high-frequencies (MIAF/DAF >0.05) in our Papuan sample which represents a small subset of the total number of variants. The current version instead begins with reporting the number of total variants retained after our filtering steps. However, after showing that the patterns of introgression do not vary between all variants and only those at common-to-high-frequency we focused main analyses on the latter. Likewise, to quantify the impact of SNPs on DNA motifs, we focused on the entire set of common-to-high-frequency variants rather than only on those segregating at frequency > 0.2 as reported in the previous submitted version. For these reasons, each section of the current version contains a greater number of variants compared to the previous version. We note that, despite this, the relative patterns of enrichment and/or proportion of variants tend to remain unchanged throughout the text relative to our previous conclusions.*

*Additionally, as we responded to the reviewers' comments, the supplementary information grew. The current version contains 9 supplementary figures, 7 supplementary tables and 3 supplementary files. Supplementary figures 1-3 were created either to justify the choice of our filtering criteria and/or to show that properties of our set of variants such as SFS and levels of background selection are not potential confounding factors in our analyses. Supplementary figure 4 was created in order to show the distribution of aSNPs and naSNPs between coding and non-coding genomic regions. Supplementary figure 5 was created to show the distribution of aSNPs across chromatin states is not affected by MIAF/DAF frequency cutoffs. Supplementary figure 6 shows that our pipeline can replicate the signals of Neanderthal introgression across chromatin states identified within Europeans. Supplementary figures 7 and 8 remained unchanged, while supplementary figure 9 compares our results to those of Findley et al.*

*We also made changes throughout the text in order to clarify our methods and discuss our findings with respect to more recent studies as suggested by reviewers. As noted above, we rewrote the subsection “Building a high-confidence set of archaic variants” of the Results section to make it more understandable. Similarly, we simplified the text contained in the “Similar patterns of introgression for Denisovan and Neanderthal aSNPs across chromatin states” subsection of the Results and its related Figure 1 in order to make comparisons between Denisovan and Neanderthal aSNPs easier to read and interpret.*

*Importantly, following one of the comments suggested by Reviewer #1, we also updated Figure 2. While Figure 2 of the past version of the manuscript reported the enrichment of TFBS-disrupting aSNPs across tissues, in the current version we extended our analyses to all variants annotated within cis-regulatory elements. We reasoned this would strengthen our findings by showing they are not biased towards a very small subset of variants. Similarly, as suggested by Reviewer #3, we changed Figure 3 in order to show not the correlation of allele frequencies between Papua New Guinea and West Indonesia but rather the F_ST_ values. We also simplified Figure 4 by removing the old panel B (i.e., the barchart containing the top significantly enriched GO terms for each ancestry) as the same information is in supplementary table 6.*

*We made changes also in the Method section. In particular, we included two new subsections, respectively describing the dataset of Neanderthal aSNPs identified within Europeans and the methodology used to calculate the Fst values between Papuans and western Indonesians. Similarly, we included a subsection (i.e., “Quantifying the levels of background selection”) describing how we assessed the levels of background selection for our set of aSNPs and naSNPs.*

*Finally, following all reviewers comments we changed the discussion of the manuscript by reporting how our results relate to previous findings on Neanderthal variants segregating within Europeans and their potential impact across chromatin states and transcription factor binding sites.*

*Reviewer #1:* In this paper, Vespasiani et al investigate the functional genetic legacy of Neandertal and Denisovan ancestry on present-day Papuans. Overall, I had some difficulty assessing the functional genetic analyses presented in this paper, largely because they fall outside my area of expertise, but also because the paper does not provide a lot of background, and in general does not justify specific filters and analyses. Thus, as currently written, following most analyses requires specific knowledge of the used packages, which I largely lack. Thus, my concerns are largely about the technical validity and clarity of the manuscript:

1. Predicting phenotypes across human populations

In the introduction, the authors point out that it remains difficult to work in non-Europeans because most reference data sets are produced in European-ancestry data sets. One well-known drawback, for example, is that genomic predictions of quantitative traits (even simple traits such as height) from one population to another have very low accuracy. Here, the authors use functional annotations made in Non-Papuans to infer the effect of archaic ancestry in Papuans. How consistent and transferable are these functional genetic categories between populations?

**The reviewer here raised an important point and we agree that more clarity was needed. While quantitative trait loci mapping is based on statistical associations between genetic variation and a molecular phenotype of interest, which are indeed highly variable across populations and depend on local recombination patterns, the definition of a chromatin state is based on a combination of multiple molecular and biochemical features. It has been shown high levels of evolutionary conservation for these biochemically-defined active non-coding regions (e.g., enhancers and promoters) both within the modern human lineage (Ward & Kellis) as well as across multiple mammalian species (Villar et al. 2016). Lastly, Naidoo et al. 2019 also showed that while the strongest levels of purifying selections are found for genomic regions located within 2.5 kb from their target genes (which are likely to contain promoter and proximal enhancer elements), regions located up 100 kb (which are more likely to contain distal enhancers) do also show signals of purifying selection. We agree that a population-specific chromatin state dataset would result in a higher accuracy when describing the potential impact of genetic mutations across functional genomic elements, however we believe that our experimental validation of the impact of some Denisovan cis-regulatory variants on gene expression to provide further support for our functional annotations.**

**We have incorporated a brief discussion on this subject into the discussion of the manuscript, as follows (line 317):**

**Applying our approach to Neanderthal aSNPs segregating within present-day Europeans [30] replicates most of our findings, as well as previously reported signals including a depletion of Neanderthal variants within enhancer-like elements [12]. Previous studies have shown that, while polygenic risk scores [51] and eQTLs [52] have limited cross-population portability, biochemically active functional regulatory elements tend to be under evolutionary constraint within the human lineage [53,54]. Thus, while our approach is contingent on functional data generated in a population that is not of Papuan ancestry our focus on functional analyses are likely to be more robust to differences across populations in linkage disequilibrium. Nevertheless, our set of SNPs, and our conclusions, represent a first step that will benefit from functional validation at scale.**

2. The filtering of SNPs is at times poorly described and justified. The authors restrict their analyses to a very small percentage of sites (<5%), following a set of stringent filtering steps. While there is some justification for these steps, my worry is that the concentration on such a small proportion of the data does introduce some biases. At the very least, it would be nice to know which step removes how many sites, and how basic properties (such as the SFS of aSNP/naSNP) change with each filtering step. For example, does restricting analyses to aSNP at frequency > 0.25 (but not having a corresponding filter for naSNP) impact the results?[IGR2]

**We have extensively rewritten that section of the methods to clarify every filtering step and provide better justifications for each decision. Ultimately, our approach is conservative, and we have erred on the side of caution, especially because the complex dynamics of introgression in ISEA, and the high distance between the Altai Denisova genome and Denisovan DNA segregating within ISEA make deconvoluting Neanderthal and Denisovan introgression a challenge.**

**We have also included a new supplementary table with the number of SNPs retained after each filtering step (supplementary table 1), a new supplementary figure showing the SFS of archaic and non-archaic SNPs before and after filtering (supplementary figure 2) and a new supplementary figure showing differences in allele frequency between archaic and non-archaic haplotypes (supplementary figure 1). See pages 36-38.**

I also have a hard time following the filters applied:

- The definitions of allele frequencies are either imprecise or wrong. For example, (p21) why is the allele frequency not calculated as (number of variant alleles) / (total number of alleles)?

**We reworded the text to clarify this point raised by the reviewer. Our decision to consider only alleles segregating on specific haplotype backgrounds when calculating allele frequencies is, as above, conservative, and geared towards controlling for incomplete lineage sorting (line 439):**

**Specifically, for naSNPs we calculated the derived allele frequency (DAF) as the number of derived alleles (relative to the EPO alignment) segregating within non-archaic haplotypes divided by the total number of chromosomes genotyped at that site. Likewise, for each aSNP, we calculated the main introgressed allele frequency (MIAF) by counting the number of observations of the most abundant allele found within archaic haplotypes divided by the total number of chromosomes genotyped at that site.**

Why is there a normalization by ancestry for allele frequencies, but not DAF and MIAF? Why do you not distinguish between homozygotes and heterozygotes?

**We apologise if this was not clear above - DAF and MIAF are normalised by ancestry, as in the definitions above and in the redrafted text.**

As defined, allele frequencies are on Papuan individuals, so a different definition must exist for sub-saharan Africans (next paragraph)

**Allele frequencies in Africa were calculated across all African samples we considered, as there are no archaic haplotypes to control for. We are aware that there are small amounts of Neanderthal DNA in some of the 1KG African populations, and we acknowledge this could lead to the loss of some Neanderthal alleles from our data.**

- the manuscript is also vague about what filtering steps were done for haplotypes, and what steps were done on SNPs, and LD was incorporated?

**We did not define the haplotypes ourselves, but rather made use of calls provided by Jacobs et al, 2019; the full methods are described there. All of our analyses are done at the SNP level, and do not incorporate LD information.**

3. Is allele frequency a confounder? Many functional categories targeted by selection are enriched at certain allele frequencies, e.g. we would expect deleterious alleles to be more rare. Due to the rather tight timing of introgression, introgressed alleles are typically much more constrained in their age (and hence allele frequency), i.e. there are no very old or very young introgressed alleles. Can you show whether there is any effect of archaic ancestry beyond allele frequency differences? If the shown results are just a byproduct of differences in allele frequency, this would be important to mention, in particular since aSNP and naSNP are differentially filtered. One possibility I could imagine this could work is if the allele frequencies were matched between naSNP and aSNP.

**We appreciate the reviewer’s suggestion about differences in allele frequency between aSNPs and naSNPs being a potential confounder in our analyses. As shown in the new supplementary figure 1, following our filtering steps aSNPs and naSNPs do indeed have different SFS. We thus randomly sampled a set of 229,541 of naSNPs in order to match the SFS of the combined set of Denisovan (140,916) and Neanderthal (88,625) aSNPs. A visual representation of the resulting SFS of both archaic and non-archaic SNPs can be found in the new supplementary figure 1c. We then used this background set of naSNPs for all our downstream comparisons.**

4. No validation of methods.

There have been several papers that have looked at the functional impact of Neanderthal ancestry in present-day Europeans (most of them cited in the paper). As the authors have their previously called haplotypes already, I understand why they chose to use a somewhat different set of filters and analyses compared to previous analyses, but one way that would raise my confidence in the presented pipeline was contrasted with previous approaches. At the very least, the results on Neanderthals should be contrasted with previous approaches in the discussion, but many of my concerns regarding adequacy of the method could be addressed by demonstrating that the presented pipeline works well and can reproduce previous results on e.g. Europeans.

**We understand the reviewer’s concerns about our findings not being replicable across other human populations. We thus analyzed a set of Neanderthal aSNPs and naSNPs identified within Europeans using our defined filtering pipeline and annotated them using the same chromatin state information. We found a large overlap between our results and previous observations by Silvert et al. 2019 and Telis et al. 2020 both in the enrichment across chromatin states (e.g., depletion from enhancers) as well as in the patterns of enrichment of Neanderthal aSNPs annotated within cis-regulatory elements across different tissues (e.g., mesenchymal and T cells). Moreover, we noted a large overlap also in our results for the set of Neanderthal aSNPs identified within Europeans and Papuans, which we believe to be consistent with the previous findings of a more uniform pattern of Neanderthal introgression across non-Africans. We thus believe our pipeline to accurately replicate some of the introgression signals already described in the literature, increasing our confidence in extending our findings to present-day Papuans. This is detailed in lines 155 and 191 of the Result section of the manuscript and can be visualized in the new supplementary figures 6 and 9.

Line 155:
Similarly to [12], we also observed a significant depletion of Neanderthal variants from Enh and EnhBiv, although we detected a significant enrichment within EnhG (Supplementary Figure S6a); however, the three states were collapsed into a single category in [12] and fewer SNPs were associated with this state than with Enh. These results not only validate our SNP filtering criteria; they also suggest that, on a genome-wide scale, different instances of admixture with archaic hominins might broadly contribute to modern human populations primarily through gene regulatory change.

Line 191:
Thus, in line with previous studies [11, 22, 31–33], our observation of a strong enrichment of Denisovan aSNPs within CREs active in immune cell types would suggest a potential contribution of these archaic variants to regulatory processes happening within immune-related cells, and by extension, to immune-related traits.**

*Reviewer #2:* Vespasiani et al. report their findings on the functional make up of genomic regions of Neanderthal and Denisovan origin in present day Papuans. As the authors mention, it is very important that previously neglected groups can also benefit from genomic studies that illuminate their past evolutions and the potential consequences for health, especially immune diseases in that case. In this case, the authors present evidence that the expression regulation of immune genes in present day Papuans has been shaped by adaptive introgression from especially Denisovan segments of DNA. Given past literature it is a very reasonable claim to make. That said, the authors will greatly strengthen their claim by adding a number of crucial controls that are currently missing from their analyses.

My main concern about the manuscript is that important controls, in the spirit of the controls implemented for example by Francesca Luca in Plos Genetics “A signature of Neanderthal introgression on molecular mechanisms of environmental responses” . It is now well known that archaic introgression is determined at the local genomic level by a combination of the local level of recombination and the local level of deleterious variation/background selection. So any over-represented immune function found by the authors could be due to this function having on average higher recombination and/or less background selection on average, rather than higher rates of adaptive introgression. This is currently a major weakness of the manuscript. It is unclear whether what the authors found is due to less background selection/higher recombination or due to genuine adaptive introgression having shaped immune regulation. The former is much more trivial and unsurprising than the latter. The authors should check what Luca et al. did to control whether or not recombination and background selection need to be controlled for. If so, the authors need to recalculate the odds ratios while matching levels of recombination (from the deCode 2019 map that is not biased by the effect of selection on LD) and levels of background selection as measured by McVicker’s B. With this done the authors will be able to make a stronger claim for pathogens in the Papuan peoples’ environments having shaped their immune genes through adaptive introgression.

**We agree with the suggestion that it is important to control for differences in the levels of background selection as a potential confounder in our analyses. We thus compared the B-statistic values for the genomic elements containing our set of Denisovan, Neanderthal and modern human variants (line 106):**

**Importantly, we found that Denisovan and Neanderthal and non-archaic variants, whether segregating at low or common-to-high-frequencies, occur within genomic regions subjected to similar levels of background selection which are also higher (i.e., corresponding to less conserved regions) than those observed for protein-coding sequences (Supplementary Figure S3a-b).**

**As shown in the new supplementary figure 3, all our variants lie within regions with similar levels of background selection. We further compared these values between low-frequency (MIAF/DAF < 0.05) and common-to-high-frequency variants (MIAF/DAF >= 0.05) and again found no differences between aSNPs and naSNPs.**

**Controlling for LD between archaic and modern haplotypes is a significant challenge, because they are drawn from two very different distributions, and defining a set of modern haplotypes of similar distribution is something we considered when we began this work but ultimately avoided as it is beyond the scope of our work (indeed, Findley et al do not control for this either). Without a doubt, the clustered nature of aSNPs relative to naSNPs will have some impact on our results, but we believe that the fact that there are two archaic ancestries in our dataset, and that the results between those differ serves as an internal control for the validity of our approach. Additionally, we have acknowledged this limitation in the discussion section (line 307):**

**While our analyses focus on a small subset of introgressed variants identified by [6], our filtering criteria ensure that we only consider variants with a high likelihood of being truly introgressed. In addition, comparing Denisovan and Neanderthal signals within the same samples serves as an internal control for difference in linkage disequilibrium and background selection between ancestries, hence providing stronger support for any eventual Denisovan- and/or Neanderthal-specific contribution to phenotypic variation in contemporary Papuans.**

Another important control that is missing and is potentially important given the results with the OAS genes and with immune over-representation overall is to control for the potential confounding effect of gene clustering. Just as the OAS genes, many immune genes are clustered together in the human genome. Therefore, it is difficult to know from the current results, whether some odds ratios are higher due to a genuine enrichment, or are spurious and due to the retained loci being clustered together. This is a pretty serious missing thing in the manuscript in its current form. The authors need to control for this, for example by counting important variants next to each other as only one variant. If they don’t do it, it could just be that the highlighted immune functions just happen to be functions that have high clustering. We can know this just from looking at where genes are in the genome, and that would weaken the claims made by the authors.

**We believe the reviewer’s concern relates to the GO enrichment analysis and, in particular, known clustering of immune genes. To clarify our analysis, GREAT assigns each variant to the nearest gene within 1 Mb of distance. This means that variants are only associated with a single gene, and clustered variants will in fact often be associated with the same gene (eg our *OAS*-associated SNPs), although sometimes. However, when performing the GO enrichment analysis, the same gene is not included multiple times, which does control for the reviewer’s concern about counting variants next to each other as a single instance. We thus believe that our method to assign variants to genes and compare the enrichment in any biological function across ancestries to be robust in accounting for eventual gene clustering.**

**We have clarified this in the methods section, line 530:**

**Importantly, by linking variants to the regulatory domain of its nearest gene, this approach does not return multiple SNP-genes associations which could occur when in presence of gene clusters.**

Minor concerns:

P3: traits of possible evolutionary advantage: misses many important citations. A few that come to mind: Gouy & Excoffier MBE 2020, Enard & Petrov Cell 2018.

**We added the suggested citations**

Figure 1 is unreadable.

**We changed figure 1 to make it more readable by showing the genome-wide patterns of enrichment of common-to-high-frequency aSNPs across chromatin states. Accordingly, we have simplified that section of the results (line 115 onwards)** **, and substantially streamlined that entire section to make it easier for readers to follow. The full results for all tests that underlie the previous figure 1 are still provided as supplementary tables.**

P7. Evolutionary importance and frequency higher than 0.2. It is very unclear and confusing what this means. It is insufficient to claim that the variants may be under positive selection. There are many more tests to evaluate such evidence, and the choice of the threshold is not justified.

**While we did not specifically test for positive selection, we believe our filtering criteria to mirror those of Sankararaman’s U statistic used to enrich for introgressed variants under positive selection, particularly a cutoff frequency of 0.2 and the absence of these variants from African individuals in the 1000 Genomes Phase 3 Panel (see Sankararaman et al. 2016 and Jagoda et al. 2021). However, we tempered our claims throughout the manuscript.**

*Reviewer #3:* The manuscript by Vespasiani and colleagues investigates the functional significance of introgressed Denisovan and Neanderthal variants in the context of transcription factor binding and gene regulation. While a few studies have analyzed Neanderthal introgressed variants and their role in gene regulation and complex trait variation in modern humans, much less is known about the potential function of non-coding variants that were introgressed from the Denisovan genomes. This is partly due to the limited availability of genomes and functional genomic data from populations where Denisovan introgression is present. This study provides a useful annotation of Denisovan introgressed variants with experimental validation of a study case for the OAS genes. The study is interesting and aligns with similar manuscripts that focus on Neanderthal introgression only, thus contributing to solve the puzzle of archaic introgression function in the human genome. Major and minor comments are listed below:

Major comments:

1. All variants that couldn't be unambiguously attributed to only Neanderthal or Denisovan origin were excluded from the analysis. These could represent archaic introgressed variants that were shared between Neanderthals and Denisovans. It would be more informative to consider them as a third group of variants to investigate and include in the analysis, alongside with the Denisovan-specific, Neanderthal-specific, and Modern Human variants. Which TFBS do they regulate, are they tissue-specific? Are they enriched in immune response regulatory regions? Considering these variants would also help understand and discuss any difference between some of the results presented here and previous studies of Neanderthal introgressed variants.

**While we agree that ambiguous aSNPs could represent shared instances between Neanderthals and Denisovans, out of the initial set of 18,518 aSNPs found to be shared between Denisovan and Neanderthal haplotypes, only 2,676 were not assigned to either ancestry. The remaining 6,098 and 9,744 were respectively assigned to Denisovan and Neanderthal ancestries based on the frequency of the variant between the two archaic haplotypes and whether the introgressed allele matched one of the two archaic reference genomes. Given such a small number of variants, we thus reasoned to remove them from the analysis rather than including them as a third archaic “source”. This is now clarified in the methods line 427:**

**Due to incomplete lineage sorting and the relatively short divergence time between Denisovan and Neanderthal lineages [2], a single archaic SNP can segregate in both Neanderthal and Denisovan haplotypes. We observed 18,518 aSNPs that segregated in both Denisovan and Neanderthal haplotypes. These variants were assigned to one of the two archaic ancestries first by considering in which archaic haplotype they segregated at higher frequencies and, then, by comparing the state of the main introgressed allele in Papuans to the Altai Neanderthal [61] or the Altai Denisovan [2] reference genomes. This resulted in 6,098 and 9,744 variants that were respectively assigned to Denisovan and Neanderthal ancestries. The remaining 2,676 variants that could not be disambiguated in this manner were labelled as ambiguous and excluded from downstream analyses.**

439

2. The manuscript correctly mentions that the genomic distribution of introgressed variants relative to functional elements could be influenced by purifying selection. The manuscript should exclude that the observed genomic distribution of introgressed variants is due to background selection, for example by comparing the B-statistic values (McVicker et al, 2009).

**As suggested also by reviewer #2, we compared the values of McVicker’s B-statistic for the genomic elements containing our set of Denisovan, Neanderthal and modern human variants. See line 106 and new Supplementary figure 3:**

**Importantly, we found that Denisovan and Neanderthal and non-archaic variants, whether segregating at low or common-to-high-frequencies, occur within genomic regions subjected to similar levels of background selection which are also higher (i.e., corresponding to less conserved regions) than those observed for protein-coding sequences (Supplementary Figure S3a-b).**

3. Intersection with eQTLs: Were only lead/top eQTLs considered for this analysis? The overlap seems very limited, which is surprising when compared with previous studies that analyzed Neanderthal eQTLs only. Given that the GTEx eQTLs are not fine mapped, it would make sense to report other significant eQTLs that overlap with introgressed variants beyond the lead SNP.

4. How do the results on TFBS disrupting variants compare with previous studies of Neanderthal introgressed variants (e.g. Silvert et al 2019, Findley et al, 2021)? Please discuss the results in the context of previous studies.

**We have substantially extended our discussion on how our results compare to the findings reported by Findley et al. 2021 with respect to TFBS-disrupting variants and their likely target genes, see line 340 and the new supplementary figure 9.**

**Third, we have characterised the impact of introgressed variants on transcription factor binding sites. A substantial proportion of aSNPs within CREs are predicted to modify the interactions between TFs and their cognate binding sites, although we do not detect an overall higher impact of aSNPs relative to naSNPs. Our data suggest that, despite aSNPs possibly disrupting individual DNA motifs, at a genome-wide level admixture with archaic hominins is unlikely to have resulted in large rewiring of transcription factor regulatory networks. While this might contrast the findings of previous studies [56], in our study we focused on families of DNA motifs (as clustered by [37]) rather than consider enrichment within individual PWMs, an approach that we believe is better suited to controlling for the typical redundancy of PWMs. This however does not exclude that aSNPs might not disrupt specific DNA motifs. Indeed, our approach also identifies Neanderthal aSNPs disrupting the PWM of TFs such as CREB1, USF1, MYB and STAT6, similarly to what reported by [56]. We also note that there is a significant (hypergeometric p lt2 · 10 −16) overlap between genes targeted by N-eQTLs in [56] and our own set of possible target genes (Supplementary Figure S9.**

5. What is the rationale behind using allele frequency correlation rather than FST to study the geographic distribution of archaic and modern human variants between the two Papuan and West-Indonesian populations? Is the FST distribution shifted for Denisovan variants compared to modern and Neanderthal variants? In general, there seems to be very little correlation in the plots despite the high R values reported.

**We agree with the reviewer’s comment and changed the analysis by calculating and reporting the distribution of the F_ST_ values between Papuans and western Indonesian populations for the set of Denisovan, Neanderthal and modern human variants annotated within cis-regulatory elements active in immune related cells. Overall, we find significantly higher Fst values for the set of Denisovan variants annotated within cis-regulatory elements active within immune-related cells compared to the other two ancestries.**

**This is now discussed in line 237 and shown as a new panel in figure 3.**

**However, in keeping with the expectation of little to no Papuan genetic ancestry in Western Indonesia only 1,535 Denisovan variants (10.8\%) in our Papuan sample are also segregating within Denisovan-like haplotypes in this additional data set (Fig. 3a). Indeed, we found significantly higher F_st_ values for the set aSNPs annotated within CREs, especially those of Denisovan ancestry(p _Wilcoxon_ < 2.2·10−6 and 0.0013, respectively for Denisovan and Neanderthal), compared to modern human naSNPs (Fig. 3b), suggesting that this set of variants might contain instances that are Papuan-specific.**

Minor comments:

1. Please improve the quality of figure 1.

**We have removed Figure 1 to a more readable format as also suggested by reviewer #2**

2. Please include a file (or link to a file in a publicly accessible location) with the rsID and genomic coordinates for the Denisovan and Neanderthal introgressed variants used in the study.

**We included supplementary file 5 and 6 respectively containing the set of Denisovan and Neanderthal cis-regulatory aSNPs together with their rsID, their putative target genes and the eventual disrupted transcription factor binding site.**
