# Supplementary material for: Denisovan introgression has shaped the immune system of present-day Papuans
Source: PLoS Genet. 2022 Dec 8;18(12):e1010470. doi: 10.1371/journal.pgen.1010470 (PMC9731433; doi:10.1371/journal.pgen.1010470)
Supplement: Attachment — Submitted filename: 20221003_archaics_in_png_response_reviewers.docx [file pgen.1010470.s020.docx]

*Reviewer #3:* This version of the manuscript by Vespasiani and colleagues presents extensive revisions to address all major comments by the three reviewers. A couple of minor points remain unaddressed, but these should be pretty straightforward to take care of:

1. Please clarify in the methods section whether any filter/significance threshold was applied on the GTEx eQTL dataset used. Were multiple SNPs per gene considered? The number of archaic alleles annotated as eQTLs has increased in this revised version, but it is unclear why

**We agree the Method section of the manuscripts currently lacks details about the definition of the cis-eQTL set of variants. We thus expanded the corresponding paragraph, including details on how we subset the list of all GTEx V8 cis-eQTLs (line 489).**

**Finally, we downloaded the list of V8 cis-eQTLs and their eGenes from the GTEx web portal (**[**https://www.gtexportal.org/home/**](https://www.gtexportal.org/home/)**) [34], retaining only the positional information for the list of significant cis-eQTLs (i.e., qval < 0.05). Variants associated with multiple genes were counted only once. The genomic coordinates for the resulting list of cis-eQTLs were then lifted over from hg38 to hg19 using liftOver from the rtracklayer R package [65]. We used this dataset to identify shared instances first only with our set of common-to-high-frequency variants annotated within putative CREs and then with our entire set of 140,916 Denisovan, 88,625 Neanderthal and 229,541 non-archaic variants. In both analyses, we retained only those variants where the reference and alternative alleles matched between the datasets.**

**We are aware about the change in number of archaic alleles annotated as eQTLs between versions. This was caused by the use of a different subset of variants between the two versions. Specifically in the last submitted version we reported the overlap between the eQTLs and our entire set of variants. We apologize for the confusion and update the manuscript by reporting both the overlap between the eQTLs and our entire set of variants (as previously reported) as well as that between the eQTLs and only the subset of common-to-high frequency variants annotated within CREs states (line 195-Results):**

**As a final test, we intersected these variants with significant cis-eQTLs (qval < 0.05) from GTEx v8 [34]. We found 59 Denisovan, 117 Neanderthal and 388 non-archaic variants (0.15%, 0.47% and 0.58% of all common-to-high-frequency SNPs, respectively) were identified as potential cis-eQTLs in at least one tissue in GTEx. We further repeated the analysis using the entire set of variants, regardless of their allele frequency, and found 132 Denisovan, 242 Neanderthal and 688 non-archaic variants (0.09%, 0.27% and 0.3%, respectively) overlapping with cis-eQTLs.**

2. Please make sure to include a file (or link to a file in a public repository) with the rs IDs and genomic coordinates for the Denisovan and Neanderthal introgressed variants used in the study, which is not limited to the ones annotated as putatively functional but includes all variants considered.

**We updated the list of supplementary tables as requested. In the current version, Supplementary Tables 2-4 respectively contain the entire list of 140,916 Denisovan, 88,625 Neanderthal and 229,541 non-archaic variants analyzed in this study. These files report the genomic position, allele frequency in our PNG sample, rsid, the putative target gene and affected PWM information for each variant.**
